# Choroid plexus volume in brain disorders: a systematic review

**DOI:** 10.1186/s12987-025-00702-4

**Published:** 2025-09-15

**Authors:** Derya Tireli, Jeppe Romme Christensen, Tina Nørgaard Munch, Nanna MacAulay, Henrik Bo Wiberg Larsson, Jonathan Frederik Carlsen, Stig Praestekjaer Cramer

**Affiliations:** 1 Functional Imaging Unit, Department of Clinical Physiology and Nuclear Medicine, Copenhagen University Hospital - Rigshospitalet, Glostrup, Denmark; 2 Department of Clinical Medicine, Faculty of Health and Medical Sciences, University of Copenhagen, Copenhagen, Denmark; 3 Danish Multiple Sclerosis Center, Department of Neurology, Copenhagen University Hospital – Rigshospitalet, Glostrup, Denmark; 4 Department of Neurosurgery, Copenhagen University Hospital, Copenhagen, Denmark; 5 Department of Congenital Disorders, Statens Serum Institut, Copenhagen, Denmark; 6 Department of Neuroscience, University of Copenhagen, Copenhagen, Denmark; 7 Department of Radiology, Copenhagen University Hospital - Rigshospitalet, Copenhagen, Denmark; 8 Centre for Functional and Diagnostic Imaging and Research, Amager and Hvidovre Hospital, Copenhagen University Hospital, Copenhagen, Denmark

**Keywords:** Choroid plexus volume, Brain disorders, Systematic review, Magnetic resonance imaging, Segmentation techniques

## Abstract

**Background:**

The choroid plexus is a highly vascularized structure located in the lateral, third, and fourth ventricles of the brain. Recent studies suggest that volumetric changes in choroid plexus volume are associated with progression in various brain diseases. Segmentation algorithms have significantly improved our ability to study choroid plexus volumetrics in relation to various pathologies. Thus, the specific purpose of this review was to describe to what extent choroid plexus volume estimation provides clinically relevant information in brain diseases.

**Methods:**

An extensive literature search was conducted across Pubmed, Embase and Cochrane databases. A comprehensive, detailed qualitative descriptive analysis, and a thorough risk-of-bias assessment were performed for the included studies.

**Results:**

Forty-eight studies were included in this systematic review in the categories of multiple sclerosis, neurodegenerative diseases, psychiatric disorders, healthy populations and a group categorized as “other” for all other brain diseases that did not fit into the other categories.

**Conclusion:**

For many of the studies included, the patients had a larger choroid plexus volume compared to healthy controls. Evidence is currently insufficient to determine whether CPV enlargement correlates with clinical severity or functional scores. The most common segmentation technique was the automatic segmentation method, followed by manual correction of the segmented choroid plexus. Thus, this review highlights the growing interest choroid plexus volume, its segmentation, and its potential as a biomarker for numerous brain diseases.

**Supplementary Information:**

The online version contains supplementary material available at 10.1186/s12987-025-00702-4.

## Introduction

### Rationale

The choroid plexus (CP) is a highly vascularized structure located in the lateral, third, and fourth ventricles of the brain. It plays an important role in brain development, maintenance, and function [1]. The CP is formed by epithelial cells arranged tightly around a stromal core that is highly vascularized and contains fibroblasts and immune cells. The CP produces cerebrospinal fluid (CSF) and is a part of the blood-CSF barrier, and thus plays a crucial role in providing nutrition, clearing metabolite waste, and conducting immune surveillance in the brain [1].

In recent years, better accessibility to magnetic resonance imaging (MRI) and enhanced segmentation algorithms have significantly improved our ability to study CP volumetrics in relation to various pathologies. Previous studies suggest that volumetric changes in the CP volume (CPV) are associated with disease progression in conditions such as Alzheimer’s disease (AD) and multiple sclerosis (MS) [2–4]. Some studies also report a correlation between CP changes and clinical worsening [3] and even suggest that CP could be a potential imaging marker for disease activity and progression [3].

Changes in CPV can be assessed by manual segmentation or with an automated tool for CP segmentation on images derived from MRI. T1-weighted and fluid-attenuated inversion recovery (FLAIR) sequences, with or without MRI contrast enhancement, are commonly used sequences to illustrate choroid plexus, due to the high image resolution, 3D coverage and superior anatomical information.

### Objectives

This review aims to summarize and clarify the current knowledge, gathered from MRI studies on CPV in various brain diseases compared to healthy controls (HC) or compared to other brain disease populations. Therefore, the specific purpose of this review was to describe to what extent CPV estimation derived from MRI provides clinically relevant information in brain diseases.

## Methods

This review was compiled and reported in accordance with the Preferred Reporting Items for Systematic reviews and Meta-analyses (PRISMA) guidelines, and the study protocol has been registered in the International prospective register of systematic reviews (PROSPERO) under the registration number CRD42024502302. The research questions were formulated using a population-intervention-comparison-outcomes-studies (PICOS) approach [5]. For further details, please see appendix 1a.

### Information sources

The literature search was conducted in MEDLINE (through the PubMed version), EMBASE Ovid and The Cochrane Library. The search date was 29 January 2024 for all three databases (Table 1). Given the volume of existing literature, the search was not updated at the time of manuscript submission.

**Table 1:** Overview of the literature search. Last searches were done 29th of august for all three databases

Database	Coverage
- Embase Ovid	1974–29.08.2024
- Pubmed	1987–29.08.2024
- Cochrane library database	−29.08.2024 (all dates)

### Search strategy

The PubMed queries with MeSH terms were (“Choroid Plexus”[Mesh] OR “Choroid plexus” OR “plexus choroid*”) AND (“Magnetic Resonance Imaging”[Mesh] OR “Magnetic Resonance Imag*” OR “NMR Imag*” OR “mr scan*” OR “mri scan*”). No additional filters were used in Cochrane database or Pubmed, however, in Embase Ovid, we provided “human” and “English language” as filters. The search string was converted to compatible versions to Cochrane and EMBASE Ovid search databases by using The Systematic Review Accelerator [6]. The search strings used in the Cochrane database and Embase Ovid are provided in Appendix 1b.

### Selection process & data collection process

Two researchers (DT and SPC) independently reviewed titles and abstracts. Duplicates, and studies that did not meet our inclusion criteria as listed previously, were excluded. In case of disagreement, final decision was made by the third researcher (JFC). Full text screening was done by first researcher, DT with assistance from SPC and JFC. Data extraction was done by DT. Title/abstract screening and full-text screening were done through Covidence software (Veritas Health Innovation, Melbourne, Australia, www.covidence.org) Data extractions were done manually in an Excel sheet.

### Data items

Any measure of CP volume and clinical assessments were eligible for inclusion.

We collected data and details on the articles, their study designs, participants and outcomes which are presented in Tables 2, 3, 4, 5, 6, and in Appendix 1c and 1d. There were no restrictions on the length of the studies.

### Study risk of bias assessment

Risk-of-bias assessments were conducted for each study using “National Institutes of Health (NIH) Quality Assessment (QA) Tool for Observational Cohort and Cross-Sectional Studies” [7].

The NIH QA tool consists of 14 criteria and is designed to help focus on key concepts for evaluating the internal validity of a study. The answer to each of the 14 criteria is Yes/No/Other (cannot determine, not reported or not applicable), which was further categorized into “low risk of bias”, “high risk of bias”, or “unclear as recommended by the NIH QA tool guide. The overall assessments are done by critical appraisal. The overall quality ratings are “good”, “fair”, or “poor” [7]. The QA assessments were done by DT, SPC and JFC.

### Effect measures

A meta-analysis was deemed not applicable, due to the vast heterogeneity of populations, measurements, and assessment scales of clinical outcomes. Therefore, a qualitative descriptive analysis was chosen as the method in this review.

### Synthesis methods

Given the amount and diversity of the studies included, we attempted to categorize the included studies into groups by disease: 1. MS, 2. Neurodegenerative diseases, 3. Psychiatric disorders,

4. Healthy populations, 5. Other brain diseases investigated. In the category of psychiatric conditions, different psychiatric conditions were included in the same group due to the smaller sample size of this category. Likewise with the group “Other”, which is a mix of different brain diseases where only one or few studies were found in the specific disease area. Some of the articles do not report all the details of the data required and although we contacted the corresponding authors, we did not receive any reply.

## Results

### Study selection

**Fig. 1:**
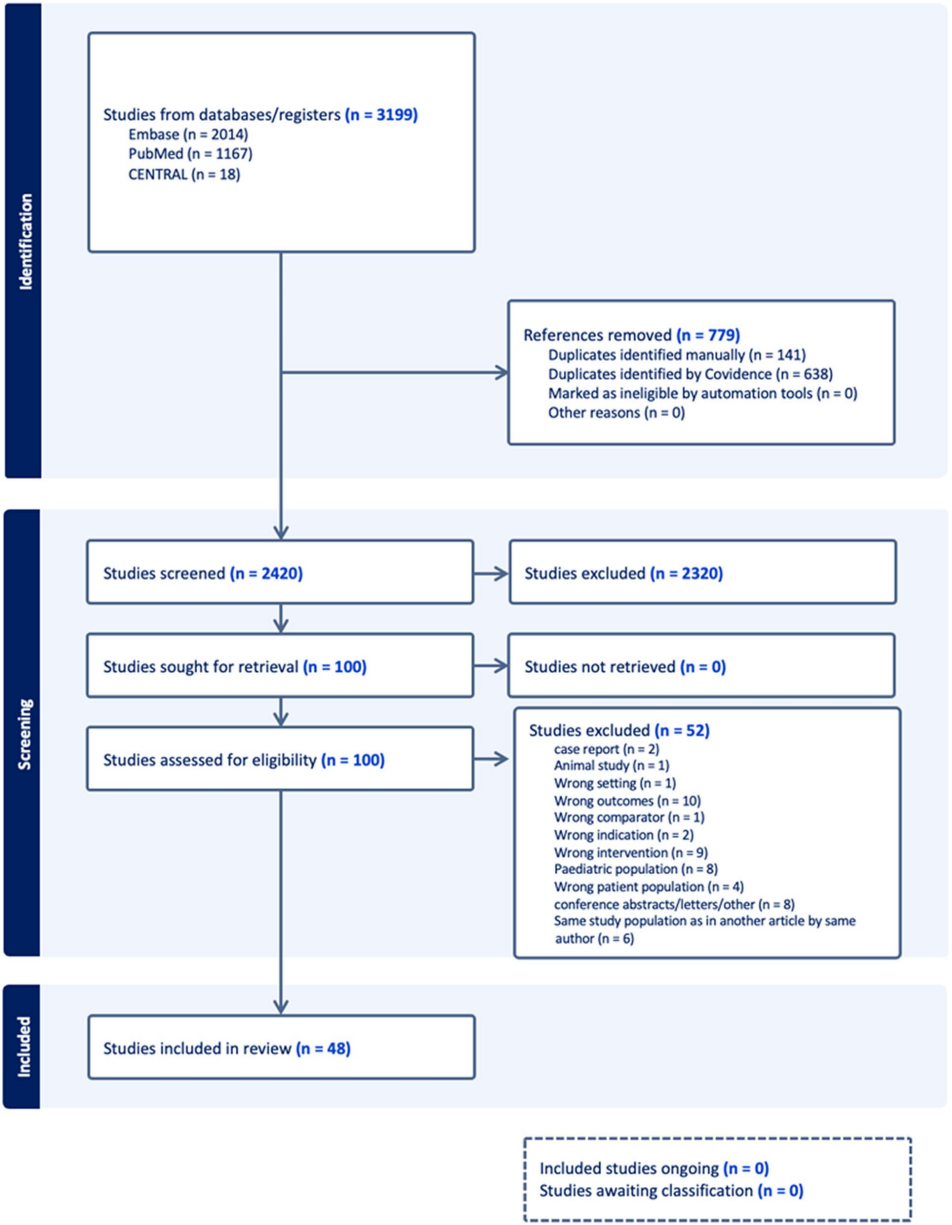
PRISMA flow diagram for the screening process

The PRISMA flowchart for the study selection process is shown in Fig. 1

In the initial search, we found 3,199 articles. After removal of duplicates, 2,320 were excluded in title/abstract screening, leaving 100 articles for full text screening. Of these 100 articles, 52 were excluded after full-text screening.

### Study characteristics and outcomes

The clinical characteristics of the 48 included studies are summarized in Tables 2, 3, 4, 5, 6. Of these, 16 investigated MS, 11 neurodegenerative diseases, 10 psychiatric disorders, 2 healthy populations, and 9 other brain diseases. Most studies were cross-sectional (CS) or retrospective (RS) in design, with 18 in each category. For all studies, relevant clinical assessment results and the relation to the CPV are presented.

**Table 2:** Clinical characteristics of studies investigating choroid plexus volume in patients with multiple sclerosis

Author, year, country	Study design	MRI acquisition, sequence for CP volume	CP assessment software used	Anatomical location of the CP segmented	Study populations (n)	CP volume results of the study populations, mean ± SD	Follow-up CP volumes, mean ± SD	Units of CP volume, TIV /NBV volume	Clinical assessment results – EDSS	Outcome
	(FU period [Years])									
Akaishi et al. 2024, Japan [8]	CS-	1.5 T, 3DT1w	Automated (FS)	LV	89 RRMS 13 SPMS 41 HC	0.09 ± 0.02** 0.11 ± 0.03** 0.08 ± 0.02**	–	%TIV	EDSS was not correlated to CP volume	Higher CPV in MS vs. HC (p < 0,05), but NS when adjusted for covariates
Bergsland et al. 2023, USA, Italy [9]	Retrospective LS (5.5y)	3 T, 3DT1w	Automated (FS) + GMM + manual correction	LV	118 RRMS 56 PMS 56 HC	2.6 ± 0.9 2.8 ± 0.9 2.3 ± 0.9	MS: 7.7% ± 30.1% HC: 10.3% ± 29.0% increase	mL / Adjusted for head size	No significance between baseline EDSS and NCPV	Higher CPV in MS vs. HC (p = 0.01) in baseline, NS when adjusted for NBV. Follow-up change NS. PMS and HC not age matched. Populations adjusted for age and gender. CPV RRMS vs. PMS NS
Chen et. al. 2022, China [10]	CS + LS	3 T, 3DT1w	Automated (FS)	LV	CS: 51 MS	Figures only	–	–	–	Higher CPV in MS vs. HC (p < 0.001), but not in NMO (p > 0.05)., Adjusted for age and gender, not brain volume. CPV change over time in MS or NMO NS
	MS: 1.4 y NMO: 1.3 y				CS: 42 NMO					
					LS: 25MS, 20NMO,56 HC					
Fleischer et al. 2021, Germany [11]	Prospective LS (4y)	3 T, 3DT1w	Automated (FS)	LV	415 RRMS 57 HC 150 CIS (CPV included in RRMS group)	Figures only	–	–	Enlarged CPV was linked to increased EDSS and lower SDMT with significance to both	Enlarged CPV in MS vs. HC (p < 0.001), adjusted for covariates, but age not strictly matched Enlarged CPV at follow-up in untreated MS + MS on DMF, but not with natalizumab
Jakimovski et al. 2024, USA [12]	LS (5y)	3 T, 3DT1w	Automated (FS) + manual rrection	LV	148 RRMS 54 PMS	2.5 ± 0.9 2.7 ± 0.9	2.6 ± 0.7 9.0% ± 33.5% 2.9 ± 1.1 10.0% ± 27.8% increase	mL/Adjusted for head size	-	CPV in RRMS vs. PMS NS both at baseline and follow-up. Not age-matched controls

Author, year, country	Study design	MRI acquisition, sequence for CP volume	CP assessment software used	Anatomical location of the CP segmented	Study populations (n)	CP volume results of the study populations, mean ± SD	Follow-up CP volumes mean ± SD	Units of CP volume, TIV /NBV volume	Clinical assessment results – EDSS	Outcome
Jankowska et al. 2023, Poland [3]	PS LS (1y)	1.5 T, 3D T1w	Automated (BrainMagic)	LV	14 RRMS w cell.therapy (treg) 18 RRMS treatment naiive 16 HC	-0.22 ± 0.05 0.20 ± 0.05	0.22 ± 0.05	%TIV	**–**	Higher CPV in MS vs. HC (p = 0.02), but not between treg and treatment naïve MS. Adjusted for covariates
Klistorner et al. 2022, Australia [13]	R LS (4y)	3 T, 3D T1w	semi-automated + manual edits	LV	49 RRMS 40 HC	0.10 ± 0.03** 0.08 ± 0.01**	**–**	%TIV	CPV/TIV ratio associated with baseline EDSS, but not at follow up	Stable CP/TIV ratio during follow-up. Enlarged CPV in RRMS vs. HC (p < 0.001). Adjusted for covariates
Klistorner et al. 2023, Australia [14]	R LS (10y)	3 T, 3D T1w	semi-automated + manual edits	LV	44 ON 50RRMS 50 HC	2.3 ± 0.5* 2.2 ± 0.7* 1.7 ± 0.3*	ON converters 2.4 ± 0.6 *	mL/Adjusted for head size	**–**	Enlarged CPV in ON and RRMS vs HC (p < 0.001), ON and RRMS no difference (p = 0.5). 23 ON who converted to RRMS after 10 y. had larger CPV vs HC (p < 0.001). Adjusted for covariates
Müller et al. 2022, Switzerland, Germany, USA, Sweden, Japan [15]	R CS (na)	3 T, 3D T1w	automatic by a deep learning algorithm	LV		(median, IQR,)	**–**	mL	**–**	Enlarged CPV in MS vs. NMO and HC (p < 0.001). No difference between NMO and HC. Adjusted for co-variates
					180 MS	1.7 (0.6) *				
					98 NMO	1.4 (0.5) *				
					94HC	1.53 (0.6) *				
Raghib et al. 2024, USA [16]	RS	3 T, 3DT1w	Automated (FS) + manual edits	LV	95 RRMS	0.7 ± 0.2 mL	**–**	TIV	Positive correlation between CPV and EDSS (p = 0.0002)	Enlarged CPV in RRMS vs. HC significantly CPV enlargement is associated with longer disease duration. Not age-matched groups, but adjusted for co
					26 HC	0.40 ± 0.09 mL				for covariates
Ricigliano et al. 2022, France [17]	RS	3 T, 3D T1w	Manual segmentation	LV	27 presympt. MS 97 MS 53 HC	0.15 ± 0.05** - 0.11 ± 0.04**	**–**	%TIV	**–**	CPV is enlarged in presymptomatic MS vs. HC (p = 0.001). No difference in presymptomatic MS vs. MS. Age and gender matched controls
Ricigliano et al. 2021, France [18]	PS	3 T, 3D T1w	Automated (FS) + manual corrections	LV	61 RRMS 36 PMS 44 HC	0.16 ± 0.05** 0.17 ± 0.04** 0.12 ± 0.04**	**–**	%TIV		Enlarged CPV in RRMS vs. HC; correlated with relapse rate (p = .005). Enlarged CPV in PMS, but no difference vs RRMS. Adjusted for covariates
Storelli et al. 2023, Italy [19]	RS	3 T, 3D T1w	1) Manual 2)Automated (FS) 3)FLAIR + T1 GMM 4) FS + GMM	LV	33 RRMS + 22 SPMS 60 HC	1)1.46 ± 0.31 2)1.38 ± 0.42 3) 1.50 ± 0.42 4) 1.80 ± 0.42 1) 1.39 ± 0.30 2) 0.74 ± 0.23 3) 1.28 ± 0.32 4) 1.24 ± 0.25	**–**	mL	Significant correlation between CPV and EDSS in MS in manual segmentation, but not in FS for manual segmentation	Enlarged CPV in MS vs. HC significantly in both methods. Not age-matched controls. Age was adjusted in CPV analysis, not in the MS vs. HC comparison

Author, year, country	Study design (FU period [Years])	MRI acquisition, sequence for CP volume	CP assessment software used	Anatomical location of the CP segmented	Study populations (n)	CP volume results of the study populations, mean ± SD	Follow-up CP volumes mean ± SD	Units of CP volume, TIV /NBV volume	Clinical assessment results – EDSS	Outcome
Tonietto et al. 2023, France [20]	LS (2-4 months + 1y)	3 T, 3DT1w	Manual segmentation	LV	59 MS 47 HC	**–**	**–**	VV	A higher periventricular remyelination was associated with a lower EDSS	CPV was associated with the periventricular failure of remyelination (p < 0.05). Not strictly age matched controls for replicated cohort
Wang et al. 2023, China [21]	RS	3 T, 3DT1w	Manual segmentation	LV	99 RRMS 60 HC	1.2 ± 0.4 mL* 0.9 ± 0.3 mL*	**–**	TIV	CPV is positively correlated with greater disability (EDSS), but NS when adjusting to TIV	Enlarged CPV in RRMS vs. HC (p < 0.001) Adjusted for covariates
Xie et al. 2024, China [22]	RS + LS (1.34y)	3 T, 3D T1w	Automated (FS) + manual corrections	LV	Baseline 65 RRMS Baseline 48 HC	0.11 ± 0.02** 0.09 ± 0.02**	0.12 ± 0.03**	%TIV	Significant correlation found between CPV and EDSS at baseline	Enlarged CPV in RRMS vs. HC (p < 0.001) Enlarged CPV at follow up (p = 0.015) Adjusted for covariates

*CS* cross-sectional study, *EDSS* expanded disability status scale, *FS* Freesurfer automated segmentation, *GMM* Gaussian mixture model. *HC* Healthy control, *LS* longitudinal study, *LV* lateral ventricle, *NS* not significant, *ON* opticus neuritis, *PMS* progressive multiple sclerosis, *PS* prospective study, *RRMS* Relapse- remitting multiple sclerosis, *RS* retrospective study, *SDMT* Symbol Digit Modalities test, *SPMS* secondary progressive multiple sclerosis, *T* Tesla, *TIV* Total intracranial volume. *Converted to mL from mm^3^ or cm^3^ **original unit converted to %TIV

**Table 3:** Clinical characteristics of studies investigating choroid plexus volume in patients with neurodegenerative diseases

Author, year, country	Study design (FU period [Years])	MRI acquisition and sequence for CP volume	CP assessment software used	Anatomical location of the CP segmented	Study populations (n)	CP volume results of the study populations, mean ± SD	Units of CP volume, TIV /NBV volume	Clinical assessment results	Outcome
Jeong et al. 2023, South Korea [2]	RS + PS	3 T, 3D T1w	Automated (FS + GMM). Manually on 20pts	LV	240 PD 80 HC	0.13 ± 0.05 ** 0.13 ± 0.05 **	%TIV	Enlarged CPV was associated with poorer frontal/executive function (p = 0.002)	No significant difference between CPV in PD vs. HC. Age matched
Jeong et al. 2023, South Korea [23]	RS	3 T, 3D T1w	Automated (FS + GMM.) Manually on 20pts	LV	322 Drug naive- early-stage PD	0.14 ± 0.06 **	%TIV	Enlarged CPV was significantly associated with higher baseline motor scores (UPDRS-III)	Enlarged CPV was significantly associated with severe baseline motor deficits + increased risk of freezing gait
Tadayon et al. 2020, USA, Spain [24]	RS	3 T, 3D T1w	Automated (FS)	LV	88 AD 119 late-MCI 127 early-MCI 208 PD 209 HC	2.9 ± 0.5 2.6 ± 0.7 2.5 ± 0.6 2.3 ± 0.7 ​ 2.5 ± 0.6	mL	–	e-MCI and l-MCI had larger CPV vs. HC (NS). CPV increased gradually with disease severity, peaking in AD CPV larger in AD vs. all groups (p < 0.05). Not age-matched groups but adjusted for covariates
Jiang et al. 2024, China[25]	PS	3 T, 3D T1w	Automated (FS + Deep learning algorithm)	–	269 MCI 228 AD HC: 110	Median (IQR) 0.15 (0.13–0.18) ** 0.18 (0.16–0.21) ** 0.12 (0.10 –0.14) **	%TIV	Enlarged CPV was correlated with decreased MMSE & MoCA, higher NPI and ADL scores	AD had the largest baseline CPV compared to the other groups (P < 0.05). Enlarged CPV was associated with decreased cognitive performance and AD CSF markers. Not strictly age matched but adjusted for covariates
Choi et al. 2022, South Korea [4]	RS + PS	3 T, 3DT1w	Automated + manual for 30pt	LV	158 early MCI 149 late MCI 147 AD	0.10 ± 0.03** 0.11 ± 0.0 ** 0.13 ± 0.04 **	%TIV	Enlarged CPV was significantly associated with severity of cognitive impairment (MMSE + CDR)	Higher CPV in patients with more severe stages (p < 0.001) of cognitive impairment
Ota et al. 2023, Japan [26]	CS	3 T, 3DT1w	Automated (FS)	–	20 AD 35 HC	0.23 ± 0.06** 0.14 ± 0.05 **	% Supratentoriel volume	The study did not focus on the MMSE results	CPV significantly higher in AD vs. HC. Not age-matched but adjusted for age and gender

Author, year, country	Study design (FU period [Years])	MRI acquisition and seq. for CP volume	CP assessment software used	Anatomical location of the CP segmented	Study populations (n)	CP volume results of the study populations, mean ± SD	Units of CP volume, TIV /NBV volume	Clinical assessment results	Outcome
Pearson et al. 2024, Australia [27]	LS	1.5 T/3 T 3DT1w	Automated (FS + GMM)	LV	115progressive MCI 338 stable MCI	Right CP: 1.29 (± 0.41) * Right CP: 1.19 (± 0.41) *	mL	The right CPV was significantly associated with lower RAVLT-I scores across all participants, NS when the two groups were analyzed separately	Significant difference for right CP with volumes larger for the progressive MCI group
Umemura et al. 2024, Japan[28]	CS	3 T 3DT1w	Automated (FS)	NS	218 MCI 1904 HC	3.40 ± 1.41 2.88 ± 1.00	mL	Higher CPV correlated with poorer MMSE scores (p < 0.01)	Enlarged CPV significantly in MCI vs. HC. Age-matched and adjusted for covariates
Assogna et al. 2023, Italy, USA, Sweden, UK [29]	Observational study	1.5 T + 3 T, 3DT1w	Automated (FS + GMM)	LV	135 bvFTD 75 PPA 60 CBS 46 PSP 82 HC	2.84 ± 0.86* 2.87 ± 0.79 * 2.71 ± 0.82 * 2.99 ± 0.90 * 1.99 ± 0.63*	mL	Higher CPV correlated with poorer MMSE scores significantly	Enlarged CPV in all subtypes vs. HC (significant). CPV showed significant correlations with cognitive/behavioural deficits and patterns of cortical atrophy. CPV NS subgroups in between. Age matched + adjusted for covariates
Jiang et al. 2023, China, Canada, USA[30]	RS	3 T, 3DT1w	Automated + manual	LV	74 bvFTD 67HC	Discovery dataset: bvFTD: 0.14 ± 0.04** Replication dataset: bvFTD: 0.12 ± 0.02** Discovery dataset HC: 0.09 ± 0.02** Replication dataset: 0.08 ± 0.02**	%TIV	In bvFTD, Enlarged CPV had a significant correlation with poorer MMSE scores	Enlarged CPV in bvFTD vs. HC (p < 0.001) Enlarged CPV was associated with global cognition scores. Not strictly age-matched, adjusted for covariates
Martinkova et al. 2023, Czech Republic, Switzerland, Sweden. [31]	RS + LS (1.07–2.18 y.)	3 T, 3DT1w	Automated + manual	LV	239 MCI 88 AD 98 Convert 188 CN	2.18 ± 0.42* 2.40 ± 0.47* 2.29 ± 0.50* 2.15 ± 0.38*	(Baseline mL)	Increased CPV over time with increasing GDS + lower MMSE-scores, but not in baseline measures	Significant annual increase in overall CPV. Largest increase in CPV in “converters”. Significant higher CPV in converters vs. CN. Significant higher CPV in baseline AD vs. CN Age-matched + adjusted for covariates

*AD* Alzheimer’s dementia, *bvFTD* behavioral variant frontotemporal dementia, *CBS* corticobasal syndrome, *CDR* clinical dementia rating, *CN* cognitive normal, *CS* cross-sectional study, *FS* Freesurfer automated segmentation, *GDS* Geriatric Depression scale, *GMM* Gaussian mixture model, *HC* Healthy control, *LS* longitudinal study, *LV* lateral ventricle, *MoCA* montreal cognitive assessment, *MCI* Mild cognitive impairment, *MMSE* mini-mental state examination, *NS* non-significant, *RAVLT-I* Rey- Auditory Verbal Learning Test, *PPA* primary progressive aphasia, *PSP* progressive supranuclear palsy, *PS* prospective study, *RS* retrospective study, *SCI* subjective cognitive impairment, *T* Tesla, *TIV* Total intracranial volume, *UPDRS-III* Unified Parkinson’s disease rating scale part III. *Converted to mL from mm^3^ or cm^3^ **original unit converted to %TIV

**Table 4:** Clinical characteristics of studies investigating choroid plexus volume in patients with psychiatric disorders

Author, year, country	Study design (FU period [Years])	MRI acquisition, sequence for CP volume	CP assessment software used	Anatomical location of the CP segmented	Study populations (n)	CP volume results of the study population, mean ± SD (mL*)	Clinical assessment results	Outcome
Bravi et al. 2023, Italy [32]	CS	3 T, 3DT1w	Automated (FS)	–	73 MDD 79 BD 72 HC	Right CP: MDD: 0.49 ± 0.17 BD: 0.50 ± 0.24 HC: 0.44 ± 0.17	–	Significantly enlarged CPV in mood disorders vs. HC Adjusted for covariates
Murck et al. 2020, Germany, USA, Slovakia [33]	RS	3 T, 3DT1w	Automated (FS) + manual	LV	16 w/ major depressive episode, 14 completed	Right CP: Non-responders: 0.60 ± 0.20 Responders: 0.51 ± 0.15	CPV correlated with clinical improvement but not significantly with treatment outcome	Enlarged CPV in non-responders’ vs. responders (NS), Enlarged CPV may be associated with poorer treatment response.​
Lavagnino et al. 2015, USA, Italy [34]	CS	1.5 T, 3DT1w	Automated (FS)	–	15 AN 15HC	–	–	CP may play a role in the neurobiology of AN Larger CPV in left side, in AN vs. HC (p < 0.001). Age matched controls
Huang et al. 2022, China, USA, Estonia [35]	CS	3 T, 3D T1w	Automated (FS)	LV	53 TRS 46 Non-TRS 66HC	1.26 (SEM 0.06) 1.26 (SEM 0.07) 0.94 (SEM 0.05)	–	Enlarged CPV in TRS and non-TRS significantly vs. HC
Zhou et al. 2020, China [36]	CS	3 T, 3DT1w	Automated (FS)	LV	79 first episode schizophrenia (FES) 41HC	1.04 ± 0.31 0.73 ± 0.20	–	Enlarged CPV was significantly associated with higher allostatic load. (cumulative physiological stress). Enlarged CPV in patients vs. HC (p < 0.0056). Adjusted for covariates
Li et al. 2021, China, USA, Estonia [37]	CS	3 T, 3DT1w	Automated (FS) + manual	–	61 TD-schizophrenia 61 Non-TD schizophrenia 74HC	TD: 2.18 ± 1.00 NTD: 1.70 ± 0.86 HC: 1.44 ± 0.84	Enlarged CPV were associated with higher orofacial AIMS scores in TD	Enlarged CPV in TD group vs. NTD and HC
Senay et al. 2023, USA, Turkey, Germany, Czech Republic [38]	CS	3 T, 3DT1w	Manual segmentation	LV	1) 41 Early-course psychosis 2) 30 chronic psychosis HC: 30 for early-course, 34 for chronic psychosis	1) Left CP: 1.2 ± 0.20 1) Right CP: 1.1 ± 0.19 2)Left CP: 0.89 ± 0.40 2)Right CP: 0.81 ± 0.42 Early-course psychosis HC: Left CP: 0.92 ± 0.19 Right CP: 0.87 ± 0.16 Chronic psychosis HC: Left CP: 0.69 ± 0.43 Right CP: 0.68 ± 0.38		Significantly higher CPV in early psychosis and a significant positive correlation between CPV and higher LV volumes in chronic psychosis
Zeng et al. 2024, China [39]	CS	3 T,3DT1w	Manually	LV	56 first-episode antipsychotic-naïve schizophrenia patients 55 HC	1.63 1.50	-	Enlarged CPV in patients vs. HC (p = 0.027) Enlarged CPV may be an inherent trait of schizophrenia
Hayasaki et al. 2024, Japan [40]	CS	3 T,3DT1w	FS	-	71 drug-naïve, first-episode MDD patients 74 HC	MDD: Right CP: 1.16 ± 0.36 Left CP: 0.95 ± 0.31 HC: Right CP: 1.01 ± 0.21 Left CP: 0.84 ± 0.18	-	Enlarged CPV in MDD vs. HC. (p-values: right ChP = 0.006, left ChP = 0.020). Not age-matched controls. Adjusted for covariates
Petzold et al. 2024, USA [41]	Retrospective CS	1.5 T, 3 T, 3DT1w	Automated (FS)	LV	89 MUD 89 HC	1.0 ± 0.03 0.84 ± 0.03	-	Enlarged CPV in MUD vs. HC (p-value < 0.001). Age matched controls. Adjusted for covariates

*AN* Anorexia nervosa, *BD* Bipolar disorder, *CS* cross-sectional study, *FES* First episode schizophrenia, *FS* Freesurfer automated segmentation, *HC* Healthy control, *LV* lateral ventricle, *MDD* major depressive disorder. *MUD* methamphetamine use disorder, *NS* not significant, *PS* prospective study. *RS* retrospective study, *T* Tesla, *TD* tardive dyskinesia, *TIV* Total intracranial volume, *TRS* treatment-resistant schizophrenia, *Converted to mL from mm^3^ or cm^3^

**Table 5:** Clinical characteristics of studies investigating choroid plexus volume in healthy populations

Author, year, country	Study design (FU period [Years])	MRI acquisition, sequence for CPV	CP assessment software used	Anatomical location of the CP segmented	Study populations (n)	CP volume results of the study population, mean ± SD	Units of CP volume, TIV /NBV volume	Clinical assess-ment results	Outcome
Alisch et al. 2021, USA [42]	CS	3 T, 3D T1w	Automated (FS) + manual correction	LV	155 Cognitively unimpaired HC	Age (per 1y- increase in age) 0.0016 ± 0.00013 Gender: 0.0070 ± 0.0052	%TIV	–	For age: CP volume (%TIV) increased non-linearly with age (adjusted for gender) (p < 0.01) For gender: No significant difference in CP volume (%TIV) in M vs. F (adjusted for age) (p > 0.1)
Eisma et al. 2021, USA [43]	CS	3 T, 3D T1w + FLAIR	Automated (Deep learning) + manual	LV	77 HC	2.81 ± 1.06*	mL	–	Age positively related to CPV (p < 0.001) and CP total blood flow (p < 0.001), negatively related to CP perfusion (p < 0.001); gender effects were significant for CPV (p < 0.001), males had larger CPV. Not adjusted for brain volume

*CS* cross-sectional study, *F* Female, *FS* Freesurfer automated segmentation, *HC* Healthy control, *LV* lateral ventricle, *M* Male, *NS* not significant, *T* Tesla, *TIV* Total intracranial volume. *Converted to mL from mm^3^ or cm^3^ **original unit converted to %TIV

**Table 6:** Clinical characteristics of studies investigating choroid plexus volume in patients with other brain diseases

Author, year, country	Study design FU period [Years])	MRI acquisition and sequence for CP volume	CP assessment software used	Anatomical location of the CP segmented	Study populationd (n)	CP volume results of the study population, mean ± SD (mL*)	Clinical assessment results	Outcome
Egorova et al. 2019, Australia [44]	LS	3 T, 3DT1w	Automated (FS)	LV	135 Ischemic stroke 40 HC	–	CPV was not correlated with NIHSS scores or lesion volumes	Enlarged CPV in stroke vs. HC, significantly. Larger CPV in chronic stages of stroke, likely occurring prior to or very early after stroke incident
Gueye et al. 2023, Italy [45]	RS	3 T, 3DT1w	Manual segmentation	LV	20 non-NPSLE 12 NPSLE 32 HC	3.17 (SE 0.12) 3.56 (SE 0.17) 2.97 (SE 0.14)	CPV enlargement is significantly associated with neuropsychiatric involvement in SLE and may serve as a reliable marker of neuroinflammation	SLE patients had significantly higher normalized CPV (nCPV) compared to HC (p = 0.023) Neuropsychiatric SLE (NPSLE) patients had significantly higher nCPV compared to non-NPSLE patients (p = 0.027) and HC (p = 0.001)
Li et al. 2023, China [46]	RS	3 T, 3DT1w	Automated (FS) + manual for a subset	LV	Cohort 1 (patients with indication for lumbar puncture): 44 peripheral neuropathy, 4 CSF leakage, 4 MND Cohort 2: 197 WMH patients	Only in figure	–	CPV was found to be higher in patients with larger WMH burden. CP enlargement was associated with impaired glymphatic clearance and larger WMH growth
Gool et al. 2023, USA, Australia.[47]	PS	3 T 3DT1w, crosschecked with T2w imaging	Manual segmentation	LV	8 NPC 8 HC	Only in figure	Overall, higher scores on the NPC-suspicion index were associated with higher expression of some biomarkers	Enlarged CPV in NPC vs. HC (p = 0.05) CP volume was positively associated with plasma PPCS levels (p = 0.05). PPCS is a biomarker assessed to understand disease status and progression. Elevated levels of PPCS can be indicative of metabolic disruptions associated with lysosomal storage disorders like NPC
Eide et al. 2020, Norway [48]	PS	3 T, 3DT1w + T2w	Automated (FS)	LV	9 iNPH 8 REF (cognitive normal)	0.88 ± 0.23 0.75 ± 0.22	NPH score (mean ± SD): REF: 15 ± 14–15, iNPH: 13 ± 11–13	No significant difference in CPV in iNPH vs. REF. After intrathecal administration of a CSF tracer, delayed clearance was observed in iNPH patients compared to REF individuals, indicating potential impaired clearance function in iNPH patients. Not age matched controls
Dai et al. 2024, China [49]	RS	3 T, 3DT1w	FS + manual correction	LV	155 ALS patients 105 HC	ALS: 2.93 ± 0.72 HC: 2.45 ± 0.78	The study found a significant correlation between CP volume and ALS-Functional Rating Scale-Revised scores (p = 0.005). As the CP volume increases, the clinical score decreases (lower scores represent more severe disability)	Enlarged CPV in ALS vs. HC (p < 0.001). Both genetic and sporadic ALS patients showed larger CPV vs. HC. The difference between genetic and sporadic ALS patients was NS
Xu et al. 2024, China [50]	CS	3 T, 3D T1w	FS	LV	116 mild WMH 62 moderate WMH 28 severe WMH 43 HC	%TIV ** 0.097 ± 0.003 0.12 ± 0.004 0.12 ± 0.004 0.082 ± 0.026	CPV was associated with decreased cognitive performance	Enlarged CPV in moderate and severe WMHs vs. HC (p < 0.001) The study suggested that glymphatic dysfunction mediates the relationship between CP volume and cognitive impairment in WMHs​
Mehan et al. 2024, USA [51]	RS	1.5 + 3 T, 3DT1w + Gd	Manual segmentation	LV	17 SIH 17 HC	1.2 ± 0.26 0.63 ± 0.31	–	Enlarged CPV in SIH vs. HC due to spinal CSF leaks (p < .0001)
Kim et al. 2024, Republic of Korea [52]	PS	3 T, 3DT1w	Automated + GMM + manuel	LV	40 OSA 38 HC	3.51 3.18	–	Enlarged CPV in OSA vs. HC (p = 0.014). CPV showed no significant correlation with polysomnographic findings or OSA severity

*ALS* amyotrophic lateral sclerosis, *CS* cross-sectional study, *FS* Freesurfer automated segmentation, *GMM* Gaussian mixture model, *HC* Healthy control, *iNPH* Idiopathic normal pressure hydrocephalus, *LS* longitudinal study, *LV* lateral ventricle, *MoCA* montreal cognitive assessment, *MMSE* mini-mental state examination, *MND* Motor neuron disease, *NPC* Niemann-Pick disease type C, *NIHSS* National Institutes of Health Stroke Scale, *NPSLE* Non psychiatric systemic lupus erythematosus, *OSA* obstructive sleep apnea, *REF* reference cohort, *RS* retrospective study, *SIH* spontaneous intracranial hypotension, *SLE* systemic lupus erythematosus, *T* Tesla, *TIV* Total intracranial volume. *WMH* white matter hyperintensity. *Converted to mL from mm^3^ or cm^3^ **original unit converted to %TIV

### Risk of bias in studies

**Fig. 2:**
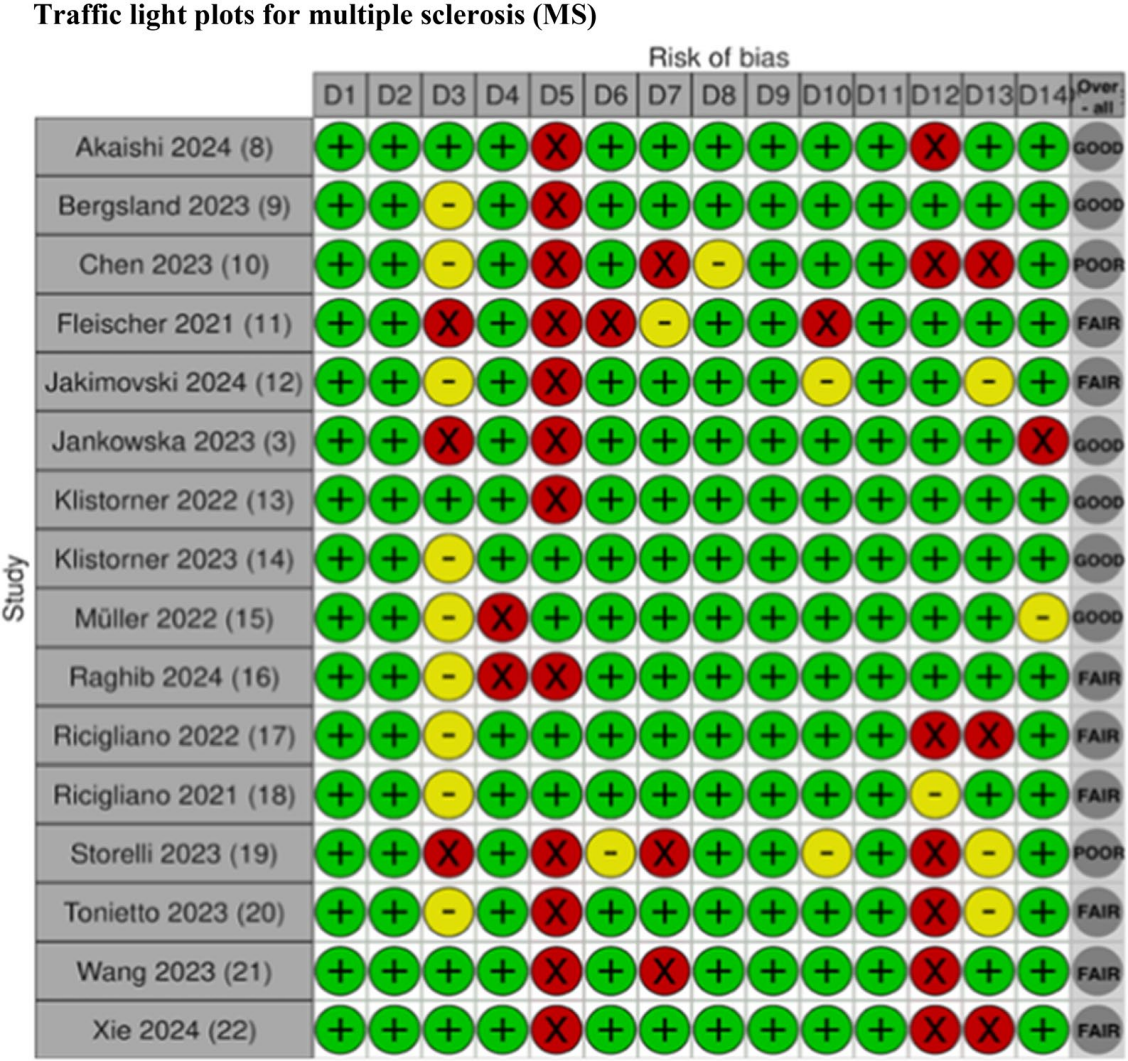
Risk of bias assessment for studies on patients with multiple sclerosis using the NIH Quality Assessment Tool

**Fig. 3:**
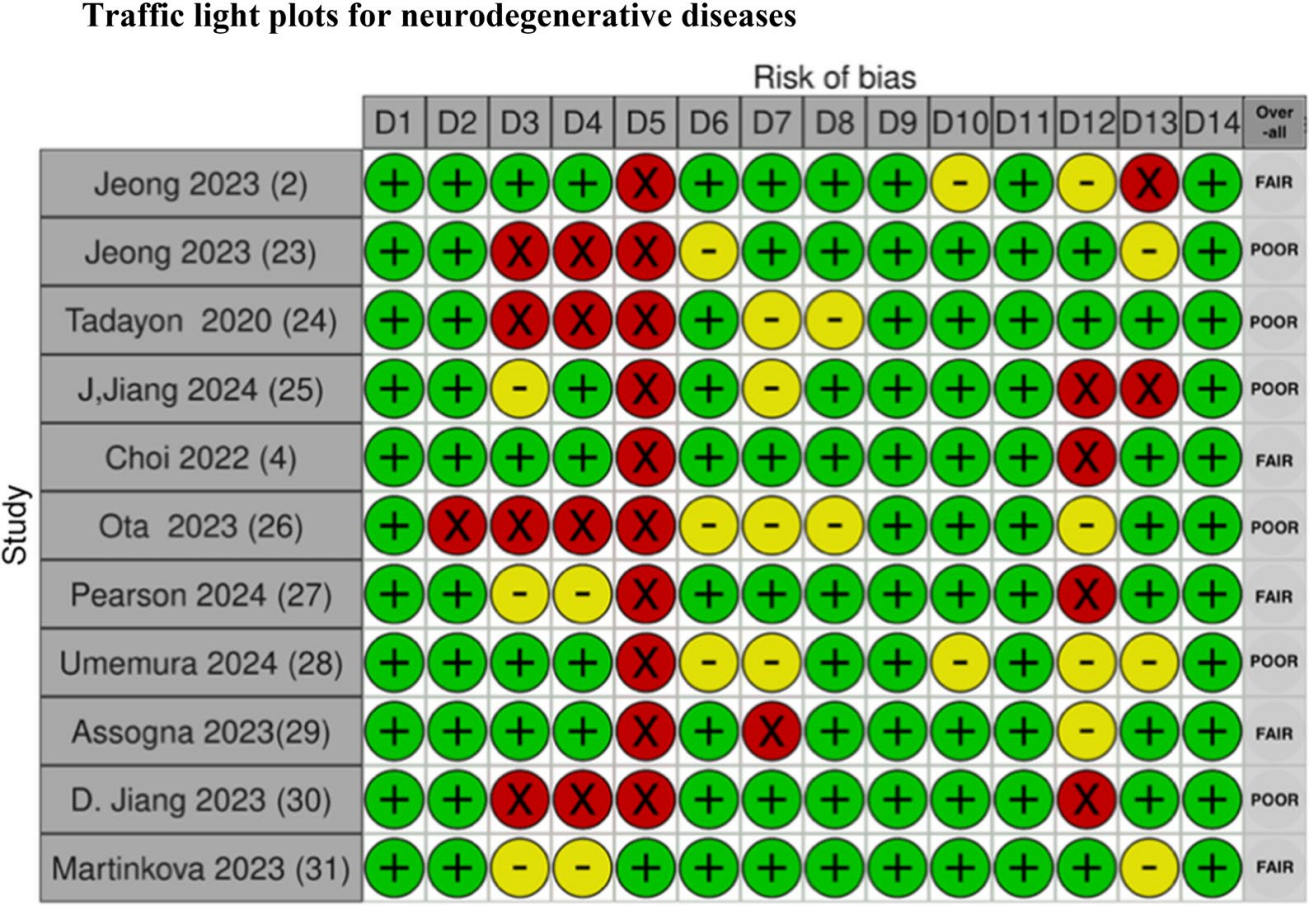
Risk of bias assessment for studies on patients with neurodegenerative diseases using the NIH Quality Assessment To

**Fig. 4:**
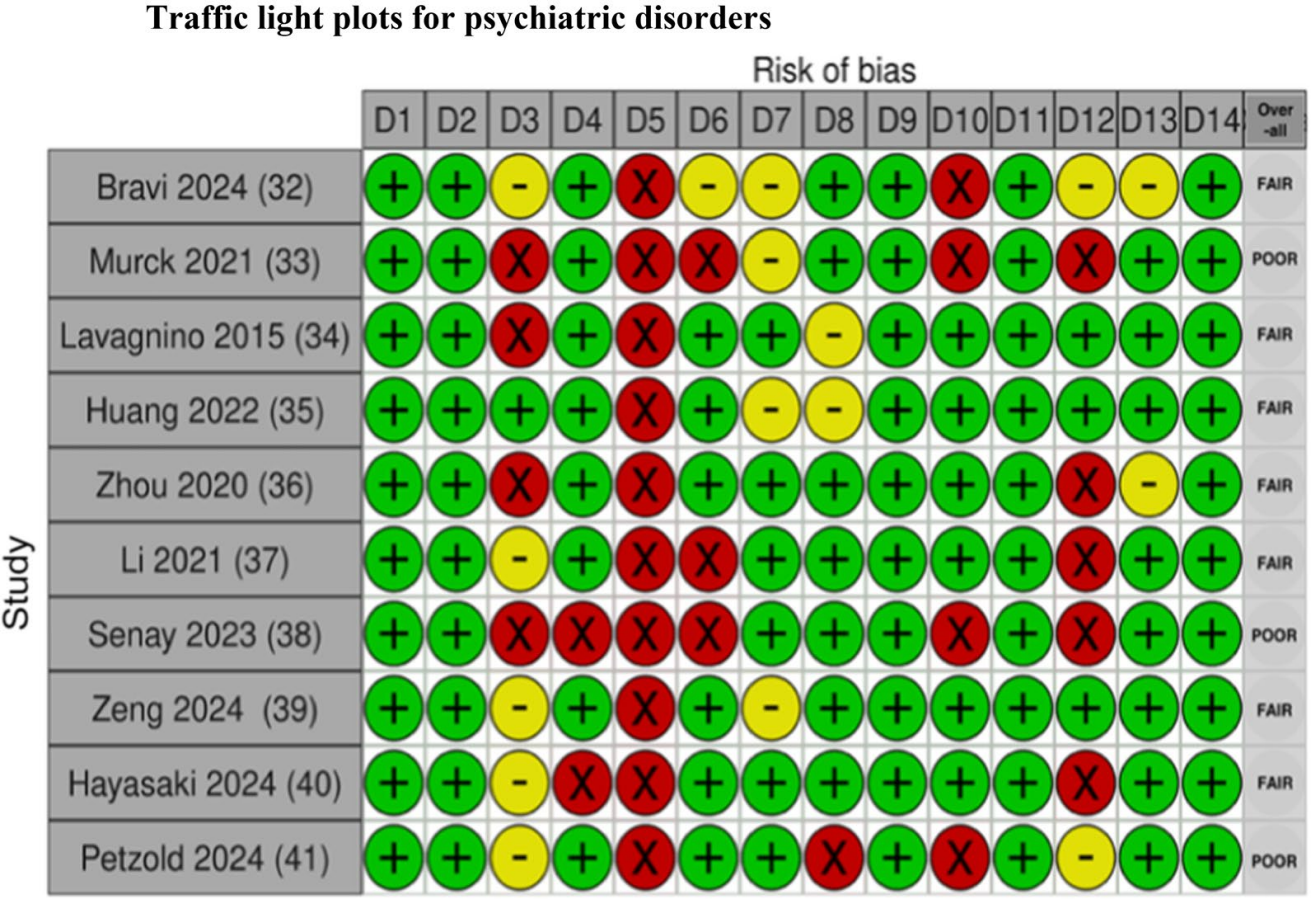
Risk of bias assessment for studies on patients with psychiatric disorders using the NIH Quality Assessment Tool

**Fig. 5:**
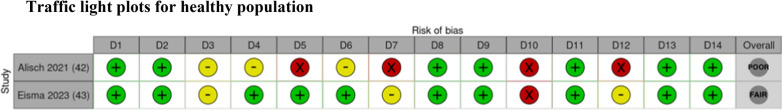
Risk of bias assessment for studies healthy populations using the NIH Quality Assessment Tool

**Fig. 6:**
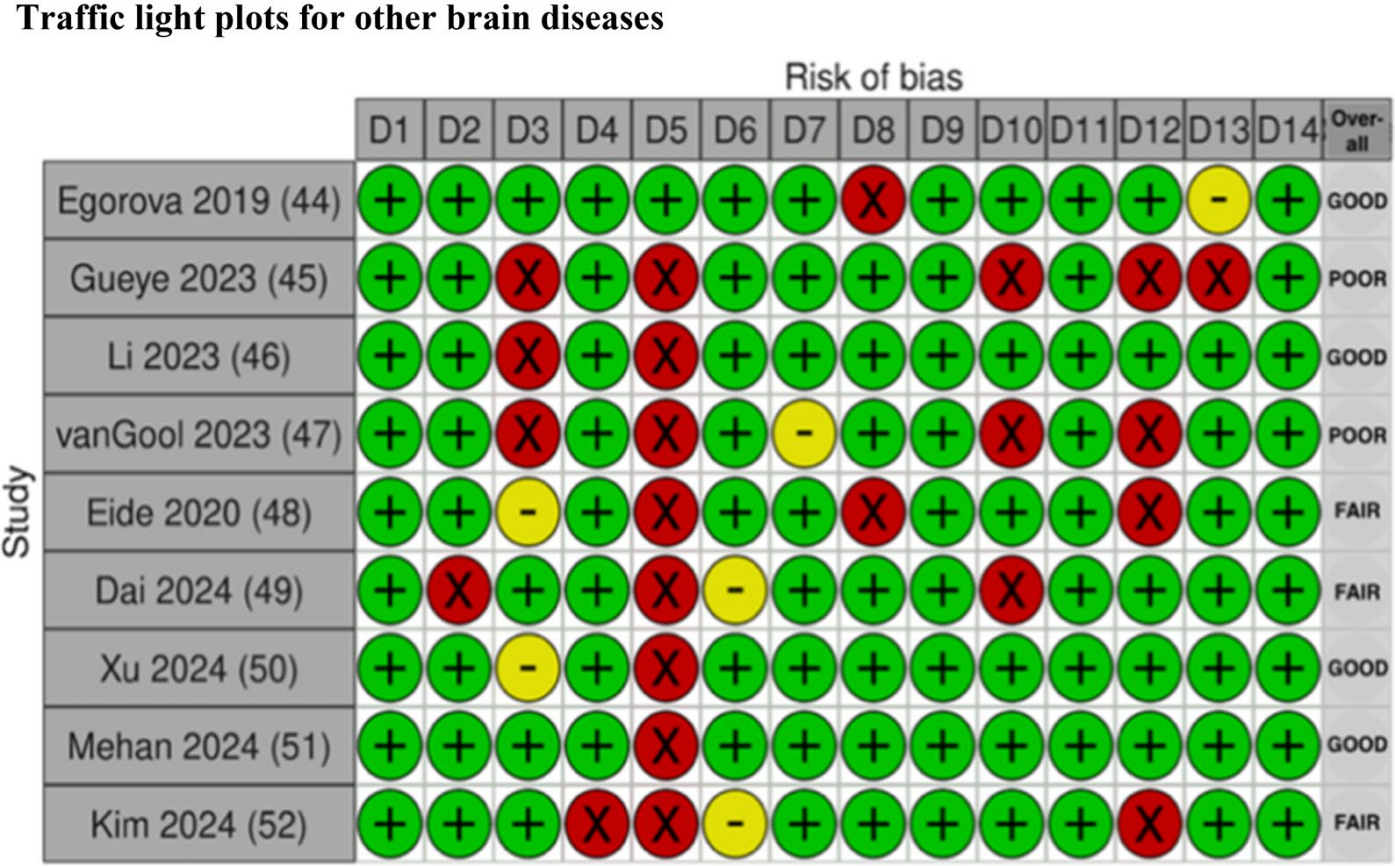
Risk of bias assessment for studies with other brain disorders using the NIH Quality Assessment Tool

The risk of bias assessments of the articles is shown in Figs 2, 3, 4, 5, 6.

The traffic light plots were made by using Shiny web app for visualizing risk-of-bias assessments [53].
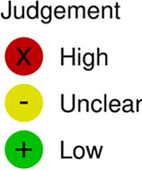


## NIH quality assessment tool criteria:

**Table:** 

D1. Was the research question or objective in this paper clearly stated?
D2. Was the study population clearly specified and defined?
D3. Was the participation rate of eligible persons at least 50%?
D4. Were all the subjects selected or recruited from the same or similar populations (including the same time period)? Were inclusion and exclusion criteria for being in the study prespecified and applied uniformly to all participants?
D5. Was a sample size justification, power description, or variance and effect estimates provided?
D6. For the analyses in this paper, were the exposure(s) of interest measured prior to the outcome(s) being measured?
D7. Was the timeframe sufficient so that one could reasonably expect to see an association between exposure and outcome if it existed?
D8. For exposures that can vary in amount or level, did the study examine different levels of the exposure as related to the outcome (e.g., categories of exposure, or exposure measured as continuous variable)?
D9. Were the exposure measures (independent variables) clearly defined, valid, reliable, and implemented consistently across all study participants?
D10. Was the exposure(s) assessed more than once over time?
D11. Were the outcome measures (dependent variables) clearly defined, valid, reliable, and implemented consistently across all study participants?
D12. Were the outcome assessors blinded to the exposure status of participants?
D13. Was loss to follow-up after baseline 20% or less?
D14. Were key potential confounding variables measured and adjusted statistically for their impact on the relationship between exposure(s) and outcome(s)?

### Results of syntheses

#### Multiple sclerosis

**CPV in MS vs. HC** Fourteen of the 16 included studies reported a significant increase in CPV in MS patients compared to HC [3, 8–11, 13–19, 21, 22]. After adjusting for covariates as age, gender, and brain volume, two of the studies found no significant difference [8, 9]. While some studies did not have age-matched controls, they did adjust the CPV analyses for age [11, 16, 19]. This is important because age affects brain and ventricle size, both of which are positively correlated with CPV [42]. Therefore, age is generally a critical confounder.

**CPV in MS vs. NMO** One study by Müller et al. found that CPV was significantly larger in MS compared to neuromyelitis optica (NMO) [15]. However, there was no significant difference in CPV between NMO and HC in the study by Müller et al. or in the study by Chen et al. [10, 15]. Chen et al. did not find significant difference between MS and NMO [10]. Furthermore, there was no significant change in CPV between baseline and follow-up in either MS or NMO [10]. Both of the studies had age- and gender-matched controls, and in addition, Müller et al. had also adjusted for total intracranial volume (TIV).

**CPV vs. clinical outcomes** Sixteen MS studies were included in this review, with 75% of the study populations being relapsing–remitting MS (RRMS). Some studies also included a second study group, such as progressive MS, two studies with neuromyelitis optica [10, 15], one with clinically isolated syndrome (CIS) [11], and one with optic neuritis (ON) [14]. Two studies did not report EDSS [3, 17]. Of the eight studies that correlated CPV with EDSS [9, 11, 13, 16, 19–22], six found a statistically significant positive correlation at baseline [11, 13, 16, 19, 20, 22]. For studies with a follow-up period [3, 9–14], CPV change over time was not significant in all, except in the studies by Jankowska et al. [3] and Fleischer et al. [11]. Jankowska et al. [3] showed that CPV increases steadily in MS, even without treatment. Fleischer et al. [11], showed a higher CPV at follow-up in untreated MS and in MS on dimethyl-fumarate (DMF), but not with natalizumab – supporting their conclusion that high-efficacy treatment can prevent CP enlargement, likely due to its role in limiting immune cell trafficking into the CNS.

### Neurodegenerative diseases

#### CPV in patients vs. HC

Eight out of 11 studies included healthy controls, and seven of these found that one or more patient groups had significantly larger CPV when compared to HC [2, 24–26, 28–31]. All had adjusted for covariates, although only three of the studies also had age-matched controls [28, 29, 31].

#### CPV in patients vs. subgroups

Four studies compared CPV of AD with MCI [4, 24, 25, 31]. Tadayon et al. also included a group of subjects with PD [24]. All these studies [4, 24, 25, 31] unanimously found that AD had a significantly larger CPV than patients with MCI and PD in baseline measurements. Choi et al. concluded that late MCI patients had significantly larger CPV than subjective cognitive impairment (SCI) and early MCI patients, but no significant difference between SCI and early MCI for CPV [4].

#### CPV vs. clinical outcomes

Eight out of eleven neurodegenerative studies used the MMSE as a clinical assessment tool [2, 4, 25, 26, 28–31], one used the UPDRS-III scale [23], one used the RAVLT-I score [27], and one study did not use any clinical assessment tools at all [24]. Out of the 11 studies, seven reported an association between CPV enlargement and lower scores in cognition tests or MMSE scores at either baseline [2, 4, 25, 28–30] or follow-up [31]. One study showed that enlarged CPV was significantly associated with severe baseline motor deficits [23]. Two of the studies did not make a correlation between the clinical assessments with CPV [24, 26].

### Psychiatric population

#### CPV in psychiatric patients vs. HC

Seven studies included healthy controls, and six of them showed significantly larger CPV in patients compared to HC [35, 37–41].

#### CPV vs. clinical outcomes

In the psychiatric field, the majority of choroid plexus volume research has focused on schizophrenia, as evidenced by five out of the ten included studies involving schizophrenia patients and utilizing PANSS scores [35–39]. In contrast, three studies focused on depression and employed HAMD scores [32, 33, 40] while one study investigated patients with anorexia [34] and another with methamphetamine use disorder (MUD) [41]. Among the five studies focusing on schizophrenia, only one specifically described the relationship between PANSS scores and CPV, concluding that there was no significant association [37]. Likewise, only one study described the relationship between HAMD scores and CPV, stating that CPV was correlated with clinical improvement but did not reach statistical significance in direct correlation with the treatment outcome [33].

### Healthy population

In the healthy populations, no clinical assessments were conducted to correlate with CPV. Both studies demonstrated a significant positive correlation between CPV and age, but found a non-linear increase in the study by Alisch et al. while the study by Eisma et al. found a linear increase with age [42, 43]. Eisma et al. [43], also reported a significant difference in CPV between males and females, finding a larger CPV in males, whereas Alisch et al. [42] did not find this difference. Notably, Eisma et al. did not normalize CPV to TIV [43], which may have influenced the findings.

### Other brain diseases

Out of nine studies in the category of other brain diseases, eight found an increase in CPV in patients compared to healthy controls, and one study by Eide et al. [48] did not. Eide et al. [48] reported no significant difference in CPV between patients with idiopathic normal pressure hydrocephalus (iNPH) and the cognitively normal population. For details of the studies included, please see Table 6.

### CP segmentation method

All studies, except three [3, 8, 34] used a 3 T scanner, and all used a T1-weighted MRI sequence to segment CPV. One study by Mehan et al. [51] used T1-weighted sequences with Gd contrast and one study by Storelli et al. [19] also utilized FLAIR to segment CPV. The study by Storelli et al. compared four segmentation methods: one manual and three different automatic approaches. Eighteen studies relied solely on automatic segmentation, primarily using FreeSurfer [3, 8, 10, 11, 24, 26–29, 32, 34–36, 40, 41, 44, 48, 50], nine studies used manual segmentation [17, 18, 20, 21, 38, 39, 45, 47, 51], and twenty-one studies used both methods, though mostly for manual corrections after the automatic segmentation or to validate the automatic segmentation techniques on smaller subgroups of patients by cross-checking manually [2, 4, 9, 12–16, 19, 22, 23, 25, 30, 31, 33, 37, 42, 43, 46, 49, 52]. Of these, three studies applied deep learning algorithms [15, 25, 43]. Automatic segmentation is less time-consuming than manual segmentation, but combining the two methods—initial automatic segmentation with subsequent manual quality assessment and correction—appears to be the current state-of-the-art approach in larger studies. This approach allows researchers to segment faster while also correcting any potential errors. Manual segmentation is commonly treated as the reference method when feasible, as seen in how the majority of the studies have manual corrections/validation after automatic segmentation. Manual segmentation allows direct boundary checking, but manual approaches have inter-rater variability and are time consuming. Automatic segmentation of CPV is particularly attractive for larger study populations, as it reduces the need for labor-intensive manual delineation, albeit potentially at the cost of reduced accuracy. In this review, we found only one study that directly compared manual segmentation with automatic segmentation [19]; therefore, evidence is limited. Storelli et al. reported that FreeSurfer only had a Dice similarity coefficient (DSC) of ~ 0.37 when compared to manual segmentation. DSC values ≥ 0.7–0.8 are typically considered good overlap for small neuroanatomical structures; thus, 0.37 indicates substantial mismatch, meaning that the automatic segmentation method by FreeSurfer is poor [19]. The automatic method proposed by Storelli et al. (ie. FLAIR + T1 GMM) significantly reduced processing time compared to FreeSurfer, and its accuracy is described as the closest method to manual segmentation. However, the DSC scores were not equally good for HC and MS patients. The segmentation accuracy was better for HC, which could be due to MS pathology making CP segmentation more difficult. This is the only study utilizing this segmentation method, and although it seems promising, more segmentation studies are needed to validate the proposed method [19].

### Risk of bias assessments

Overall, there is limited information regarding sample size justification and power description in most of the studies, as seen in the risk of bias (ROB) figures, domain five (D5) (see Figs 2, 3, 4, 5, 6). The studies included in this review demonstrate the heterogeneity in sample size, ranging from as few as eight patients [47] to as many as 415 patients [11]. This wide variability reflects a broad spectrum of study designs, and therefore, it can also complicate the interpretation and comparability of findings across studies. Without a power calculation, there is no assurance that the sample sizes are sufficient or that the significant results are reliable, especially in the smaller studies. This variation emphasizes the need for standardized guidelines and sufficient sample sizes to ensure reliable and generalizable conclusions.

## Discussion

There has been a vastly increasing interest in CP segmentation in recent years for many brain diseases. This interest continues to grow and expand, with more diseases being studied in relation to CP.

The studies presented in this review used a T1-weighted (T1w) MRI sequence for segmentation of the CPV. Only Mehan et al. [51] used T1w sequences with MR contrast agents in patients with spontaneous intracranial hypotension compared to HC. However, the study does not clarify the risk of mis-segmentation of non-choroid plexus structures due to contrast. The authors only state that they excluded choroid plexus cysts and xanthogranulomas. They also found that their patient population had a significantly larger CPV compared to HC.

The study by Storelli et al. [19] used FLAIR + T1 sequences as part of one of their four segmentation methods. Since CP appears hyperintense on FLAIR, using FLAIR + T1 helped separate CP more accurately from CSF. One study, by Eide et al. used T2-weighted (T2w) images in addition, but only as a correction tool in cases where the automatic segmentation tool made errors. The study does not mention whether adding T2-weighted images as a correction tool improved segmentation [48].

When performing CP volume segmentation, T1w, T2w, and FLAIR sequences each have advantages and disadvantages. However, T1w MRI is generally the preferred choice for CP segmentation because it provides high anatomical resolution, making the CP easier to separate from the ventricles, as it appears hyperintense compared to CSF. Furthermore, the T1w sequence can be easily used in segmentation algorithms such as FreeSurfer automatic segmentations. Therefore, the T1w sequence is the standard for volumetric analysis and is frequently used in these types of studies. In T2w sequences, the CP appears isointense to surrounding brain tissues, making it harder to segment accurately. With FLAIR, the CP boundaries can become blurred due to signal attenuation, making it less reliable for precise structural delineation. Similarly, T1w sequences with MR contrast agents are useful for finding pathology in MRI, but for standard CP volume segmentation, T1w without Gd contrast is the better choice.

The obtained CP volumes vary from study to study. One could argue that T1w MRI is perhaps the preferred method for segmentation, but whether automatic or manual segmentation is superior is difficult to determine. Furthermore, some studies use the CP/TIV ratio as the reported measure, while others rely solely on cubic volume measurements. The ratios were converted to %TIV in this review, as seen in Tables 2, 3, 4, 5, 6. Therefore, it is challenging to establish a reference value for a HC CPV or to determine the extent of CP enlargement in brain diseases. However, it can be concluded that, in most studies, HCs have a smaller CPV compared to individuals with brain diseases and that clinical severity scores might correlate with CPV enlargement. In MS, some of the studies with a follow-up period found no significant difference between baseline and follow-up CPV [9, 10, 12, 13]. These observations imply that the pathophysiology behind the CP enlargement must occur early in the disease course in MS patients. It should be noted, however, that most available studies have relatively small sample sizes and use employ heterogeneous imaging and segmentation methods, which limits the strength and generalizability of their conclusions. This review suggests that CP enlargement is not specific to any particular neurological disease discussed here, and while it may serve as a general biomarker for pathology, it does not appear to have sufficient specificity to predict or distinguish individual brain disorders.

The studies included in this review have several limitations. The CP of the third and fourth ventricles is harder to segment due to their limited visibility. One limitation across all studies, is the location of CP segmentation. In most cases, segmentation is stated to be from the lateral ventricles, while some studies do not specify where it was segmented from. More accurate segmentation methods, including the third and fourth ventricles, along with higher-resolution structural images, may provide a better evaluation of CP volume. Another limitation was the variability in how CP volume was reported. Due to the large differences in CP measurement results as mentioned, it was not possible to conduct a meta-analysis.

Regarding clinical outcome scores, six [11, 13, 16, 19, 20, 22] out of eight of the MS studies that investigated the correlation between EDSS and CPV, and seven of the 11 studies on neurodegenerative diseases, found that CPV enlargement was significantly associated with clinical severity scores in baseline measurements [2, 4, 23, 25, 28–30]. For the remaining two categories, psychiatric conditions and other, there is not enough data on clinical assessments. Therefore, we can conclude that there is currently some evidence for the association between disease severity scores and CPV enlargement, however more sufficiently powered studies are needed to establish this relationship.

Other general limitations are differences between studies in age and gender composition. Both studies focusing on healthy populations, examined gender differences in CP volume. Alisch et al. [42] reported no statistically significant difference between males and females, while Eisma et al. [43] found that males had significantly larger CP volumes compared to females. However, only Alisch et al. performed normalization to intracranial volume, which indicates that gender differences are mainly driven by a larger intracranial volume in males. This could complicate direct comparison of CPV between different pathologies, since the gender distribution is highly heterogeneous, e.g. MS affecting more females, as opposed to e.g. PD affecting more males. This highlights the importance of normalization by intracranial volume, which should minimize this bias, and improve generalization of results between studies.

Only two studies have directly examined healthy populations, and both reported a positive correlation between CPV enlargement and increasing age. Alisch et al. found a non-linear increase in CPV with age, while Eisma et al. found a linear increase. Both results indicate that CPV increases with age. Since Alisch et al. investigated a much larger study population (n = 155) as opposed to Eisma et al. (n = 77), one could speculate if the non-linear increase in CPV with age describes a more accurate physiological changes in CPV with increasing age. Such a strong age effect implies that even small mismatches in age distribution between groups can bias results. If study and control groups are not age-matched, one might over- or underestimate the disease effects. Therefore, it is crucial to perform exact age- and gender matching for control groups and adjust very carefully these covariates in statistical analyses to avoid misleading results. In this review, all studies except one [23] have included a control group, however strict age-matching between groups is not always possible, e.g. in cases comparing different disease stages MCI vs. AD [4]. This leaves the possibility of small residual age related CPV differences, even when age adjustment is done. In these cases, it is enlightening to include healthy controls in similar age categories to delineate the age effect.

We noted a marked difference in CP volume among healthy controls across published MRI studies. This is presumably due to differences in age, gender, segmentation methods, outcome measures of CPV and MRI scanners between cohorts. Together, these factors widen the spread of HC values across studies even when methods appear similar. MRI-derived CP volumes and post-mortem CP weights are broadly comparable in magnitude, but they are not directly interchangeable. Pathology weights depend on dissection boundaries, blood content, and fixation, while MRI volumes depend on contrast and segmentation rules. Any conversion between weight and volume requires density assumptions and will carry error. Pathology measurements are useful, but interpretation and comparisons should be done cautiously.

Further studies on healthy populations are needed to establish a reliable and well-defined baseline for CP volume, to allow the determination of normal reference ranges for CP volume with age. In order to CP volume can be used in the daily hospital setting, more research is needed to clarify how gender, age, or brain size influences CPV and how these factors should be accounted for.

Two studies [9, 12] reported the percentage change of CPV during long-term follow-up. Both Jakimovski et al. and Bergsland et al. had a large sample size, but only Bergsland et al. had healthy controls. Both had a long-term follow-up for 5 [12] and 5.5 years [9]. Interestingly, Bergsland et al. found that patients had a 7.7% increase and HC had a 10% increase in CPV during follow-up, while Jakimovski et al. found a 9 and 10% increase in CPV for patients. Although this data is rather limited, it may indicate that CPV changes over time, are governed more by a physiological age-related increase, rather than pathology. Bergsland et al. also investigated patients who progressed in their disability status over the follow-up and compared them with those who remained stable and found no significant difference between the groups [9].

Most of the studies were cross-sectional, so they did not investigate treatment response, which might be observable in longitudinal studies. Only two studies compared treated patients: the study by Jankowska et al. which compared RRMS patients receiving cellular therapy with treatment-naïve MS patients [3], and the study by Huang et al. [35], which compared treatment-resistant schizophrenia (TRS) patients with non-treatment-resistant schizophrenia (NTRS) patients and HC. However, while the studies found significant differences between study populations and healthy controls, they did not find significant differences between the two RRMS groups or between the schizophrenia patient groups. Studying treatment response in relation to CP volume is important because it helps determine whether CP volume changes are a consequence of disease progression, a marker of treatment effects, or a predictor of treatment response. If CP volume decreases with treatment, it may indicate a reversible disease process or an effect of medication. Therefore, further longitudinal studies are needed to investigate treatment response.

## Conclusion

This review highlights the growing interest in choroid plexus volume as a potential biomarker for a range of brain diseases. Numerous studies have reported CPV enlargement across diverse neuropathologies compared with healthy controls, suggesting that it is not specific to a single condition. Evidence is currently insufficient to determine whether CPV enlargement correlates with clinical severity or functional scores. In clinical practice, CPV enlargement may indicate the presence of disease, but the absence of standardized measurement protocols and the variability of published findings limit its current use in diagnostic settings.

From a policy perspective, establishing standardized protocols for CP segmentation and volume reporting would improve comparability across studies. Differences in MRI methodologies, segmentation techniques, and normalization methods make it difficult to determine reference values for CP volume. Guidelines on the best imaging protocols and segmentation methods could enhance reproducibility and reliability, enabling CP volume to be integrated into clinical workflows.

Future research should examine whether CP volume changes with treatment and whether it predicts disease progression. Investigating its role in immune function, the blood-CSF barrier, and waste clearance may uncover new therapeutic targets. Integrating CP volume with biomarkers of neuroinflammation, glymphatic dysfunction, and CSF proteins could strengthen its clinical relevance.

## Supplementary Information

Additional file 1.

## Data Availability

No datasets were generated or analysed during the current study.

## Abbreviations

AD: Alzheimer’s dementia
ALS: Amyotrophic lateral sclerosis
AN: Anorexia nervosa
BD: Bipolar disorder
BIMC: Blessed information memory concentration
bvFTD: Behavioral variant frontotemporal dementia
CBS: Corticobasal syndrome
CDMS: Clinically definite multiple sclerosis
CDR: Clinical dementia rating
CIS: Clinically isolated syndrome
CN: Cognitively normal
CSF: Cerebrospinal fluid
CP: Choroid plexus
CS: Cross-sectional study
CDR: Clinical dementia rating
DMF: Dimethyl fumarate
EDSS: Expanded disability status scale
F: Female
FTLD: Frontotemporal lobar degeneration
FS: FreeSurfer automated segmentation
GMM: Gaussian mixture model
Gd: Gadolinium
HAMD: Hamilton depression rating scale
HC: Healthy control
ICV: Intracranial volume
iNPH: Idiopathic normal pressure hydrocephalus
LS: Longitudinal study
LV: Lateral ventricles
M: Man/men/male
MCI: Mild cognitive impairment
MDD: Major depressive disorder
MMSE: Mini-mental state examination
MoCA: Montreal cognitive assessment
MRI: Magnetic resonance imaging
MUD: Methamphetamine use disorder
nFL: Neurofilament light chain
NIHSS: National institutes of health stroke scale
NMO: Neuromyelitis optica spectrum disorder
NPC: Niemann-Pick disease type C
NS: Not significant
NPSLE: Non psychiatric systemic lupus erythematosus
NR: Not reported
OCT: Optical coherence tomography
ON: Opticus neuritis
OSA: Obstructive sleep apnea
PANSS: Positive and negative syndrome scale
PMS: Progressive multiple sclerosis
PPA: Primary progressive aphasia
PPCS: N-palmitoyl-O-phosphocholineserine
PS: Prospective study
PSP: Progressive supranuclear palsy
RAVLT-I: Rey- auditory verbal learning test
REF: Reference cohort
RRMS: Relapsing–remitting multiple sclerosis
RS: Retrospective study
SCI: Subjective cognitive impairment
SDMT: Symbol digit modalities test
SLE: Systemic lupus erythematosus
SIH: Spontaneous intracranial hypotension
SPMS: Secondary progressive multiple sclerosis
T: Tesla
TBV: Total brain volume
TD: Tardive dyskinesia
TIV: Total intracranial volume
TRS: Treatment-resistant schizophrenia
UPDRS-III: Unified Parkinsons disease rating scale part III
WMH: White matter hyperintensity
y: Year

## Declarations

Ethics approval and consent to participate.

Not applicable.
