# Supplementary material for: Choroid plexus volume in brain disorders: a systematic review
Source: Fluids Barriers CNS. 2025 Sep 15;22:92. doi: 10.1186/s12987-025-00702-4 (PMC12439388; doi:10.1186/s12987-025-00702-4)
Supplement: Supplementary file 1 — Additional file 1. [file 12987_2025_702_MOESM1_ESM.docx]

**Appendix 1**

1. **PICOS approach:**

*Population*: We included adult patients (age ≥18 years) within the neurological, neurosurgical, and psychiatric fields. We excluded patients with primary choroid plexus (CP) disease such as choroid plexus xanthogranulomas, choroid plexus papillomas and tumors.

*Intervention:* We included patients who had undergone T1-weighted magnetic resonance imaging (MRI) with or without gadolinium (Gd) enhancement or/and fluid-attenuated inversion recovery (FLAIR) imaging performed with a minimum field strength of 1.5 Tesla. We only included studies that reported CP volume measurements.

*Comparators:* We compared the study populations with each other within the same intervention. The primary comparators/controls were healthy controls (HC). Secondary comparators were diseases within the neurological field compared to each other, as well as the subphenotypes within each disease.

*Outcome:* The primary outcome was the difference in choroid plexus volume (CPV) ratio between groups (e.g. between patients and healthy controls), between subgroups of patients (e.g. relapsing-remitting and progressive multiple sclerosis), or between patients with disease progression and without disease progression (e.g. as measured by the expanded disability status scale (EDSS) in multiple sclerosis (MS)). The CPV ratio was defined as the ratio of the CP volume (e.g. in mm^3^) to the total intracranial volume (TIV), derived from volumetric MRI (T1w or FLAIR) by either manual segmentation, automatic segmentation, or both.

*Studies*: We included all types of published MRI-studies investigating the connection between CPV and clinical information (prospective and retrospective studies, with or without cross-sectional analyses). The articles had to be written in English. We excluded case reports, reviews, conference abstracts, letters, studies with pediatric population, animal studies, unpublished work, and studies with overlapping data; however, studies from same author but with different relevant comparisons with CPV were still mentioned briefly in this review. We also excluded studies where the outcome – the CPV – were not measured.

1. **Search strings:**

For cochrane database:

([mh "Choroid Plexus"] OR "Choroid plexus" OR ("plexus" NEXT choroid*)) AND ([mh "Magnetic Resonance Imaging"] OR ("Magnetic Resonance" NEXT Imag*) OR ("NMR" NEXT Imag*) OR ("mr" NEXT scan*) OR ("mri" NEXT scan*)) = 18 I search manager

Embase Ovid: *limited to human studies, and english language.*
(exp "Choroid Plexus"/ OR "Choroid plexus" OR "plexus choroid*") AND (exp "Magnetic Resonance Imaging"/ OR "Magnetic Resonance Imag*" OR "NMR Imag*" OR "mr scan*" OR "mri scan*")

1. **Data items:**

We collected data on:
*Report:* Title, author, year, country, study design, purpose of the study, funding sources.

*Participants:* Population groups, inclusion and exclusion criteria for study participants, number of patients and controls, and demographic data.

*Outcome:* Intervention characteristics, CP assessment tools, CP volume with chosen units, the anatomical location of the segmentation in the brain of the CP, clinical assessments scores.

Some of these data were not reported in this review to maintain focus. Data will be available upon request.

The following clinical assessment scores were extracted if they were reported in the article:

Multiple Sclerosis (MS): Expanded Disability Status Scale (EDSS)

Parkinson Disease (PD): Mini Mental State Examination (MMSE), Unified Parkinson's Disease Rating Scale – Part III (UPDRS-III score)

Dementia: MMSE, **Clinical Dementia Rating** (CDR) and subtypes, Montreal Cognitive Assessment (MoCA), Geriatric Depression Score (GDS) and Rey Auditory Verbal Learning Test – Immediate (RAVLT-I)

Mood-disorders: Hamilton Depression Rating Scale (HAMD)

Anorexia Nervosa (AN): Body Mass Index (BMI), Body satisfaction score, Drive for thinness

Schizophrenia**: Positive and Negative Syndrome Scale** (PANSS)

1. **Table supplements**

**Table S2.** Clinical characteristics of studies investigating choroid plexus volume in patients with multiple sclerosis

| **Author, year, country** | **Age, mean ± SD for study population (years)** | **Sex of study population** | **Age, mean ± SD for control group (year)** | **Sex of control group** | **MRI study intervention** | **Clinical assessment scores – EDSS, median (IQR)** |
| --- | --- | --- | --- | --- | --- | --- |
| Akaishi et al. 2024,  Japan (8) | RRMS: median 39 (IQR 33-45)  SPMS: median 45  (IQR 43-52) | 78.7% F  61.5% F | HC:  median 41 (IQR 27-51) | 65.9% F | MRIx1 | RRMS: 2.0 (1.0–3.0) SPMS: 6.0 (4.5–7.0). |
| Bergsland et al. 2023, USA, Italy (9) | RRMS: 44.4 ± 10.9 PMS: 54.0 ± 7.2 | 70% F  82% F | HC:  45.4 ± 13.7 | 70% F | MRI at baseline + 5.5y follow up | Baseline:  RRMS 2.0 (1.5–2.6) PMS 6.0 (4.0–6.5).  Follow-up  RRMS 2.5 (1.5–3.5) PMS 6.5 (4.5–6.6) |
| Chen et al. 2022,  China (10) | CS:  MS: 40.67 ± 14.08 NMO: 40.48 ± 13.70  LS:  MS: 38.8 ± 11.11  NMO: 41 ± 13.78 | CS:  63% F  93% F  LS:  56% F  95% F | HC: 37.38 ± 14.29 | 55% F | MRIx2.  interval range  median  MS 1.37y NMOSD 1.25y | MS 2.50 (2.00)  NMO 3.00 (1.50). |
| Fleischer et al. 2021, Germany (11) | Main cohort:  36.1 ± 10.8,  Replication cohort: 33.4 ± 9.6 | 70% F   74% F | HC: 29.9 ± 9.2 | 54% F | MRI imaging annually for 4 y for patients; 1 for HC | Main cohort:  1.5 ± 1.41 (0 - 7.5)  Replication cohort:  1.5 ± 0.98 (0 - 4.5) |
| Jakimovski et al. 2024, USA (12) | RRMS: 44.1 ± 10.6 | 71.6% F | PMS: 55.3 ± 7.9 years | 83.3% F | MRI at baseline + 5y follow up | Baseline:  RRMS: 1.5 (1.5–2.5), PMS: 6.0 (4.0–6.5)  Follow-up:  RRMS: 2.0 (1.5–3.5), PMS: 6.5 (4.0–6.5) |
| Jankowska et al. 2023, Poland (3) | 36.16 ± 8.42  (all MS) | 60% F | HC: 38.06 ± 7.09 | 56 % F | Treg group: MRI at +3, +6 and +12 months.  Treatment naïve group: MRI at +6 and +12months.  MRI x 1 for HC. | - |
| Klistorner et al. 2022, Australia (13) | RRMS: 41.4 ± 9.1 | 59% F | HC: 37.4 ± 7.7 | 60 % F | RRMS: MRI at 0, 12 and 60months follow up.  HC: MRIx1 | Baseline:  1.0 (range 0–5)  Follow-up:  1.0 (range 0–6). |
| Klistorner et al. 2023, Australia (14) | ON: 33.9 ± 8.9  RRMS: 38.2 ± 9.3 | 70%F  66% F | HC: 36.0 ± 7.4 | 70% F | ON: MRI at baseline, 1,3,6 + 12m follow up after ON onset. RRMS and HC: MRI x 1. | ON baseline: 3 (range 0–4)  ON 10 years follow-up: 0 (range 0–2) RRMS baseline: 1 (range 0–4) |
| Müller et al. 2022, Switzerland, Germany, USA, Sweden, Japan (15) | MS: *median* 46.6 (IQR 15.3)  NMO: *median* 54.5 (IQR 18.5) | 70% F  88% F | HC: *median* 46.1 IQR (22.3) | 64% F | MRIx1 | MS 2.5 (3.5),  NMO 3.5 (2.8) |
| Raghib et al. 2024,  USA (16) | RRMS: 41.7 ± 10.24 | 65.3% F | HC: 33.4 ± 11 | 61.5% F | MRIx1 | RRMS: 2.00 (1.00–4.00) |
| Ricigliano et al. 2022, France (17) | Presymptomatic MS:  42 ± 11  MS: 42 ± 12 | 53% F (all MS) | HC: 42 years ± 14.4 | 53% F | MRIx1 | - |
| Ricigliano et al. 2022, France (18) | RRMS: 37± 10  PMS: 50 ±11 | 51% F  50 % F | HC: 39 ± 14 | 52% F | MRIx1 | RRMS = 2 (range 0-6), PMS= 6 (range 2,5-7,5) |
| Storelli et al. 2023,  Italy (19) | MS: 46.8 ± 10.2 | 56% F | HC: 36.1 ± 12.6 | 55% F | MRIx1 | All MS: 2.5 (range 1.0-6.0) |
| Tonietto et al. 2023, France (20) | Original MS cohort: 32.3 ± 5.6  Replication MS cohort: 49.4 ± 10.8 | 60% F  55% F | Original HC cohort: 31.6 ± 6.4  Replication HC cohort: 40.8 ± 10.7 | 62.5% F  57.5% F | MRI at baseline + follow-up (2-4 months for the original cohort, 1 year for the replication cohort). | Original Cohort: 2 (range 0–6)  Replication Cohort, 4.5 (range 1–7.5). |
| Wang et al. 2023,  China (21) | RRMS: 33.62 ± 10.01 | 81% F | HC: 36.60 ± 9.58 | 72% F | MRIx1 | RRMS: 1.0 (2) |
| Xie et al. 2024,  China (22) | RRMS: 35.40 ± 12.07 | 63.1% F | HC: 31.25 ± 9.99 | 64.6 % F | MRIx2 (20 RRMS)  MRIx1 (HC + 45 RRMS) | 3.0 ± 1.5. |

*Abbreviations: CS: cross-sectional study, EDSS: expanded disability status scale, F: female, FS: Freesurfer automated segmentation, GMM: Gaussian mixture model, HC: Healthy control, LS: longitudinal study, LV: lateral ventricle, ON: opticus neuritis, PMS: progressive multiple sclerosis, PS: prospective study. RRMS: Relapse- remitting multiple sclerosis, RS: retrospective study, SDMT: Symbol Digit Modalities test, SPMS: secondary progressive multiple sclerosis, T: Tesla, TIV: Total intracranial volume. *Converted to mL from mm^3^ or cm^3^ **original unit converted to %TIV.*

**Table S3.** Clinical characteristics of studies investigating choroid plexus volume in patients with neurodegenerative diseases

| **Author, year, country** | **Age, mean ± SD (year)** | **Sex of study population** | **Age, mean ± SD for control group (year)** | **Sex of control group** | **MRI study design** | **Clinical assessment results (mean ± SD)** |
| --- | --- | --- | --- | --- | --- | --- |
| Jeong et al. 2023, South Korea (2) | For all PD patients: 67.89 ± 7.88 | 50.4% F | HC: 66.64 ± 9.41 | 55.0% F | MRIx1 at baseline  Clinical follow-up period 7.35 ± 2.27y | MMSE (PD): 26.95 ± 2.31. HC: Normal MMSE. UPDRS-III (PD): 21.98 ± 10.36. Cognitive composite scores higher in HCs than in PD patients across all domains. |
| Jeong et al. 2023, South Korea (23) | Drug naive- early-stage PD: 64.14 ±9.72 | 53% F | 0 | 0 | MRIx1 | Baseline UPDRS-III score: 23.26 ± 10.50 |
| Tadayon et al. 2020, USA, Spain (24) | AD: 74.6 ± 8.0  PD: 61.4 ± 9.5 | 44% F 31% F | HC (AD cohort): 73.4 ± 6.3 HC (PD cohort): 60.2 ± 11.3 | AD cohort, HC: 51 % F PD cohort, HC: 29% F | MRIx1 | There is no direct mention of clinical scores in the study. |
| Jiang et al. 2024, China(25) | MCI: 64.83 ± 7.61  AD: 70.04 ± 8.96 | 62.45 % F 58.33% F | HC: 60.44 ± 7.13 | 56.36% F | MRIx1 | MMSE (median (IQR)):  MCI: 26 (24-28) AD: 16 (9-22) MoCA (median (IQR)):  MCI:22 (19-24)  AD:11(5-16) |
| Choi et al. 2022, South Korea (4) | SCI: 69 ± 8  early MCI: 70 ± 9  late MCI: 73 ± 8  AD: 76 ± 8 | 73% F 75% F 72% F  71% F | 0 | 0 | MRIx1 | MMSE: SCI: 28, eMCI: 27, lMCI:25, AD:20  p<0.001. |
| Ota et al. 2023, Japan (26) | AD: 70.8 ± 9.0 | 70% F | HC: 64.6 ± 7.8 | 57% F | MRIx1 | MMSE: AD: 20.8 ± 3.1. HC: 29.2 ± 1.1, p<0.001 |
| Pearson et al.  2024, Australia (27) | progressive MCI: 3.76 ± 7.62 | 46.09% F | Stable MCI: 72.70 ± 7.55 | 40.8% F | MRIx1 | RAVLT-I Scores: pMCI: 30.55 ± 8.73. sMCI: 37.33 ± 10.81 |
| Umemura et al. 2024, Japan (28) | MCI: Median age 72y (IQR 69–76) | 45 % F | HC: Median age 69y (IQR 66–73) | 63% F | MRIx1 | MMSE Score (median (IQR))  MCI: 25 (19–29)  HC: 29 (19–30) |
| Assogna et al. 2023, Italy, USA, Sweden, UK (29) | bvFTD: 65.4 ± 8.0 PPA: 65.5 ± 8.9 CBS: 64.9 ± 7.8  PSP: 72.5 ± 6.7 | 44% F  60% F  45% F  41% F | HC: 51.9 ± 15.0 | 61% F | MRIx1 | MMSE:  bvFTD: 21.40 ± 5.99, PPA: 17.89 ± 8.36  CBS: 23.13 ± 5.90, PSP: 24.30 ± 5.47,  HC: Not reported. |
| Jiang et al. 2023, China, Canada, USA (30) | Discovery dataset. bvFTD: 63 ±7.73, Replication dataset: bvFTD:60.72 ± 5.97 | Discovery dataset: 62% F Replication dataset: 31% F | HC Discovery dataset: 60.31 ± 8.31 HC Replication dataset: 60.03 ± 7.15 | Discovery dataset: 62% F Replication dataset: 55% F | MRIx1 | MMSE: bvFTD (Discovery dataset): 17.65 ± 6.03, HCs: 28.66 ± 1.88 MMSE: bvFTD (Replication dataset): 24.79 ± 4.31, HCs: 29.26 ± 0.86 |
| Martinkova et al. 2023,  Czech Republic, Switzerland, Sweden (31) | Baseline:  MCI: 71.88 ± 7.39  AD 74.09 ± 8.03  Convert ± 73.53 7.27. | MCI 45% F  AD 43% F Convert 45% F | CN: 72.62 ± 6.57 | 56% F | MRIx2 | MMSE at baseline (median (IQR)) CN=29.00 (29.00, 30.00), MCI 29.00 (27.00, 29.00), AD 23.00 (21.00, 25.00), convert 28.00 (26.00, 29.00) p <0.001 |

*Abbreviations: AD: Alzheimer dementia, bvFTD: behavioral variant frontotemporal dementia, CBS: corticobasal syndrome, CDR: Clinical dementia rating, CS: cross-sectional study, F: female, FS: Freesurfer automated segmentation, GMM: Gaussian mixture model, HC: Healthy control, LS: longitudinal study, LV: lateral ventricle, MoCA: montreal cognitive assessment, MCI: Mild cognitive impairment, MMSE: mini-mental state examination, NS: non-significant, RAVLT-I: Rey- Auditory Verbal Learning Test 1, PPA: primary progressive aphasia, PSP: progressive supranuclear palsy, PS: prospective study, RS: retrospective study, SCI: subjective cognitive impairment, T: Tesla, TIV: Total intracranial volume. UPDRS-III: Unified Parkinson’s disease rating scale part III. *Converted to mL from mm^3^ or cm^3^ **original unit converted to %TIV.*

**Table S4.** Clinical characteristics of studies investigating choroid plexus volume in patients with psychiatric disorders

| **Author, year, country** | **Age, mean ± SD (year)** | **Sex of study population** | **Age, mean ± SD for control group(year)** | **Sex of control group** | **MRI study design** | **Clinical assessment results (mean ± SD)** |
| --- | --- | --- | --- | --- | --- | --- |
| Bravi et al. 2023,  Italy (32) | MDD: 49.3 ± 9.4 BD: 47.3 ± 12.0 | MDD: 62% F  BD: 68% F | HC: 48.9 ± 9.8 | 62,5% F | MRIx1 | HAMD- Scale: MDD: 21.4 ± 7.1  BD: 19.5 ± 6.5 |
| Murck et al. 2020, Germany, USA, Slovakia (33) | Major depressive episode: 37 ± 12 | 50% F | 0 | 0 | MRIx1 | HAMD-21: 23.8 ± 4.8. |
| Lavagnino et al. 2015, USA, Italy (34) | AN: 20 ± 4 | 100% F | HC: 22 ± 3 | 100% F | MRIx1 | BMI: HC 21.4 ± 2.4, AN 15.9 ±1.0 p <0.001 Drive for thinness: HC 2.3 ±2.8,  AN 12.8 ±7.1 p<0.001 Body dissatisfaction: HC 7.9 ±6.9,  AN 12.8 ± 6.9 p=0.061 |
| Huang et al. 2022, China, USA, Estonia (35) | TRS: 46.08 (SEM: 1.39)  NTRS: 46.71  (SEM 1.58) | TRS: 30% F  NTRS: 35% F | HC: 42.62 ± 1.32 | 41% F | MRIx1 | PANSS total score: TRS 84.98 ± 1.92 NTRS 49.54 ± 1.59  MCCB total score: TRS 39.54 ± 1.68 NTRS 45.20 ± 1.66, HC 58.06 ± 1.05 |
| Zhou et al. 2020,  China (36) | FES: 27.2 ± 7.6 | 52% F | HC: 29.8 ± 6.4 | 49% F | MRIx1 | PANSS total score: 78.1 ± 12.4 Positive subscale: 22.5 ± 5.2 Negative subscale: 17.6 ± 6.3 General psychopathology: 37.8 ± 6.7 |
| Li et al. 2021, China, USA, Estonia (37) | TD group: 48.43 ± 9.21, NTD group: 46.21 ± 8.94 | TD group: 26% F  NTD group: 43% F | HC: 48.15 ± 8.10 | 39% F | MRIx1 | PANSS baseline total score:  NTD 65.52 ± 16.00, TD 67.64 ± 14.25  p= 0.393,  AIMS: TD 7.86 ± 3.57, NTD 0. |
| Senay et al. 2023,  USA, Turkey, Germany, Czech Republic (38) | Early-course psychosis: 21.44 ± 4.01  Chronic psychosis: 33.07 ± 9.48. | 37% F  37% F | Early-course HC: 25.03 ± 4.52 Chronic psychosis HC: 32.50 ± 9.10 | 60% F.  38% F. | MRIx1 | PANSS total score:  Early-course psychosis: 47.38 ± 9.81 Chronic psychosis: 57.13 ± 15.38. |
| Zeng et al. 2024,  China (39) | Schizophrenia: 24.70 ± 6.53 | 59% F | HC: 24.80 ± 2.28 | 69% F | MRIx1 | PANSS Total score: 95.45 ± 12.46. |
| Hayasaki et al. 2024, Japan (40) | MDD: 52.58 ± 16.16 | 59% F | HC: 35.12 ± 12.32 | 35% F | MRIx1 | MDD, HAMD-17 Total Score: 22.28 ± 6.36 |
| Petzold et al. 2024,  USA (41) | MUD: 36.0 ± 7.5 | 38% F | HC: 34.9 ± 6.9 | 38% F | MRIx1 | Beck Depression Inventory (BDI) scores: MUD group: 8.7 ± 8.6, HC group: 3.1 ± 5.5 p < 0.001 |

*Abbreviations: AN: Anorexia nervosa, BD: Bipolar disorder, CS: cross-sectional study, F: female, FES: First episode schizophrenia, FS: Freesurfer automated segmentation, HC: Healthy control, LV: lateral ventricle, MDD: major depressive disorder, NS: non-significant, PS: prospective study, RS: retrospective study, T: Tesla, TD: tardive dyskinesia, TIV: Total intracranial volume, TRS: treatment-resistant schizophrenia, *Converted to mL from mm^3^ or cm^3^*

**Table S5.** Clinical characteristics of studies investigating choroid plexus volume in healthy populations

| **Author, year, country** | **Age, mean ± SD (year)** | **Sex of study population** | **MRI study design** | **Clinical assessment results** |
| --- | --- | --- | --- | --- |
| Alisch et al. 2021,  USA (42) | 53.3 ± 21.3 | 42% F | MRIx1 | Blessed Information Memory  Concentration (BIMC) score (score ≥4 excluded) + Clinical Dementia Rating (CDR) (score ≥0.5 further review for cognitive test). |
| Eisma et al. 2021,  USA (43) | 47.8 ± 18.2 | 55% F | MRIx1 | 0 |

*CS: cross-sectional study, F: Female, FS: Freesurfer automated segmentation, HC: Healthy control, LV: lateral ventricle, T: Tesla, TIV: Total intracranial volume. *Converted to mL from mm^3^ or cm^3^ **original unit converted to %TIV.*

**Table S6.** Clinical characteristics of studies investigating choroid plexus volume in patients with other brain diseases

| **Author, year, country, disease** | **Age, mean ± SD (year)** | **Sex of study population** | **Age, mean ± SD for control group (year)** | **Sex of control group** | **MRI study design** | **Clinical assessment results (mean ± SD)** |
| --- | --- | --- | --- | --- | --- | --- |
| Egorova et al. 2019, Australia,  Ischemic stroke (44) | IS: 67.41 ± 13.01 | 30% F | HC: 68.65 (6.64) | 38% F | MRI at baseline, 3 months, and 12 months post-stroke | NIHSS baseline median score of 2 (range 0-15). |
| Gueye et al. 2023,  Italy, Systemic lupus erythematosus (45) | All SLE: 39.9 ± 12.4 | 81% F | HC: 40.4 ± 12.6 years | 81% F | MRIx1 | - |
| Li et al. 2023, China, Cohort 1: (patients with indication for LP) peripheral neuropathy, CSF leakage, MND  Cohort 2: WMH patients (46) | Cohort 1: 59 ± 13 | 50% F | Cohort 2:  63 ± 9 | 45% F | Cohort 1: baseline MRI (before LP) + 39 hours after intrathecal gadolinium injection. cohort 2: Baseline MRI + median follow-up time of 1.4 y | - |
| Gool et al. 2023,  USA, Australia,  Niemann-Pick disease type C (47) | NPC: 36.00 ± 14.27 | 38% F | HC: 36.13 ± 14.23 | 38% F | MRIx1 | - |
| Eide et al. 2020,  Norway,  Idiopathic normal pressure hydrocephalus (48) | iNPH:  68.3 ± 9.5 | 11% F | REF (cognitive normal): 38.4 ± 17.3 | 75% F | MRI before and through 24h after intrathecal administration of gadobutrol | NPH score  REF: median 15 (IQR 14-15)  iNPH: median 13 (IQR 11-13) |
| Dai et al. 2024,  China,  Amyotrophic lateral sclerosis (49) | ALS: 57.1 ± 9.6 | 39% F | HC: 57.3 ± 7.9 | 48% F | MRIx1 | Amyotrophic Lateral Sclerosis Functional Rating Scale-  Revised (ALSFRS-R): 40.3 ± 3.7 |
| Xu et al. 2024, China,  White matter hyperintensity (50) | Mild WMH: 66.2 ± 7.3  Moderate WMH: 71.9 ± 7.3  Severe WMH: 69.4 ± 7.4 | 59 % F  (all patients) | HC: 61.6 ± 7.8 | 70 % F | MRIx1 | MMSE:  HC: 28.12 ± 1.52  Mild WMH: 27.49 ± 2.27  Moderate WMH: 27.05 ± 2.77  Severe WMH: 26.14 ± 4.29 |
| Mehan et al. 2024, USA,  Spontaneous intracranial hypotension (51) | SIH: 45.1 ± 14.5 | 47 % F | HC: 45.3 ± 14.3 | 47 %F | MRIx1 | Bern score (a score to predict the probability of CSF leak. 0 - 9 = low to high probability)  SIH: 7.8, ± 1.4 HC: 0 |
| Kim et al. 2024, Republic of Korea, Obstructive sleep apnea (52) | OSA: 62.7 ± 13.9 | 38 % F | HC: 61.7 ± 6.8 | 26 % F | MRIx1 | Median Epworth Sleepiness Scale score: 4 (IQR 3–8). |

*Abbreviations: ALS: amyotrophic lateral sclerosis, CS: cross-sectional study, F: female, FS: Freesurfer automated segmentation, GMM: Gaussian mixture model, HC: Healthy control, iNPH: Idiopathic normal pressure hydrocephalus, IS: ischemic stroke, LP: lumbar puncture, LS: longitudinal study, LV: lateral ventricle, MoCA: montreal cognitive assessment, MMSE: mini-mental state examination, NPC: Niemann-Pick disease type C, NIHSS: National Institutes of Health Stroke Scale, NPSLE: Non psychiatric systemic lupus erythematosus, OSA: obstructive sleep apnea, REF: reference cohort, RS: retrospective study, SIH: spontaneous intracranial hypotension, SLE: systemic lupus erythematosus, T: Tesla, TIV: Total intracranial volume, WMH: white matter hyperintensity. *Converted to mL from mm^3^ or cm^3^ **original unit converted to %TIV*
